# Tracing the eastward dispersal of the house mouse, *Mus musculus*

**DOI:** 10.1186/s41021-015-0013-9

**Published:** 2015-08-01

**Authors:** Hitoshi Suzuki, Lyudmila V. Yakimenko, Daiki Usuda, Liubov V. Frisman

**Affiliations:** Laboratory of Ecology and Genetics, Graduate School of Environmental Earth Science, Hokkaido University, Sapporo, 060-0810 Japan; Ecological Department, Vladivostok State University Economics and Service, Vladivostok, 690014 Russia; RIKEN, Bioresource Center, Tsukuba, 305-0074 Japan; Institute for Complex Analysis of Regional Problems FEB RAS, Birobidzhan, 679016 Russia

**Keywords:** Anthropogenic movement, Phylogeography, Prehistoric colonization, *Mus musculus wagneri*

## Abstract

Here we describe recent advances in our understanding of the natural history of the house mouse, *Mus musculus*, with a focus on the genetic characteristics of the home territories and how this relates to prehistoric eastward movements from the predicted source areas. Recent studies of mitochondrial and nuclear gene sequences provide insight into the ancient divergence of the three subspecies groups, *M. m. castaneus* (CAS), *M. m. domesticus* (DOM), and *M. m. musculus* (MUS), with inferred natural habits (homelands) in central (Iran, Afghanistan, Pakistan, and India), western (western Iran), and northern (central Asia) areas, respectively. Our mitochondrial DNA and nuclear gene analyses indicate that only one local lineage of CAS extended its range via historical rapid expansion at two different times to Southeast Asia and East Asia, including Japan and southern Sakhalin. This is suggestive of a rapid range expansion of CAS out of its homeland, perhaps associated with the spread of agricultural practices in Asia. The subspecies group MUS now occurs in a large portion of northern Eurasia from eastern Europe in the West to the Japanese Islands in the East, including Uzbekistan, Kazakhstan, southern Siberia, northern China, and Korea, showing divergent patterns in terms of *Mus musculus* genetics, particularly in relation to nuclear gene sequences, allozymes (e.g., hemoglobin), morphological characteristics, and cytogenetic C-banding patterns. In this review article, we explain the complex spatial patterns of MUS. We postulate that two historical dispersal events took place, from two different source areas, and tentatively assign the taxon names “*musculus*” and “*wagneri*” to the two populations, which are associated with distinct genetic modules.

## Introduction

This review addresses the eastward movements of subspecies of the house mouse, *Mus musculus*, from their respective source areas. We focus on *M. m. castaneus* (CAS) and *M. m. musculus* (MUS), the natural histories of which are seldom discussed, compared to the remaining major subspecies group, *M. m. domesticus* (DOM). We propose revised hypotheses regarding three important topics: 1) a candidate site of origin for *Mus musculus*, 2) the long-distance dispersal of CAS, and 3) the long-range dispersal of MUS from two postulated source areas in the northeastern part of Eurasia. These insights contribute towards our knowledge of the genetic architecture of the house mouse and our understanding of the prehistoric and historic human-assisted movements of wild mice across the Asian part of the Eurasian continent.

### Identification of the homeland based on genetic analysis

**Fig. 1:**
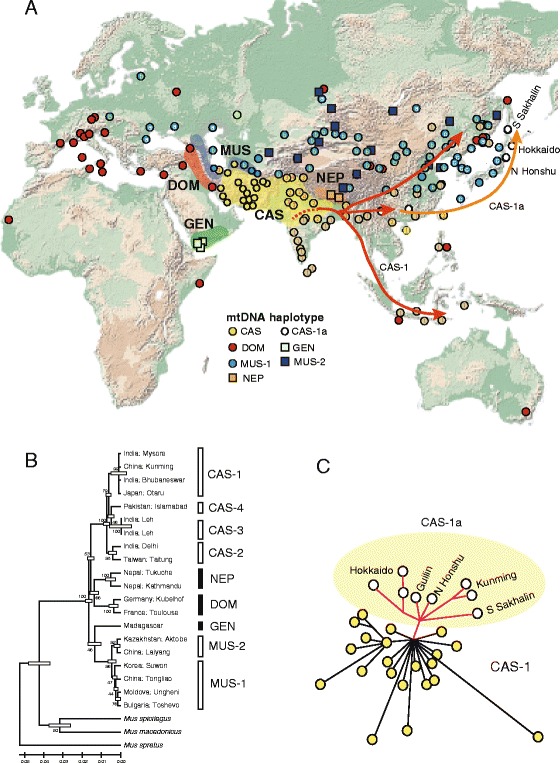
Geographic distribution of the five mtDNA phylogroups of the house mouse *Mus musculus* [3, 5], with their tentatively predicted homelands (**a**). Five distinct lineages are represented by the following taxon names: *M. m. musculus* (MUS), *M. m. domesticus* (DOM), *M. m. castaneus* (CAS), *M. m. gentilulus* (GEN), and the Nepalese lineage (NEP). Further subdivision of the MUS lineages into two others (MUS-1, MUS-2), and the MUS-1 sublineage, in turn, into three others (MUS-1a, MUS-1b, MUS-1c) was proposed in a previous study [5]. The sub-group types of MUS-1 and CAS (CAS 1–4) are shown in circles with letters or numerals, respectively [5]. A maximum likelihood tree based on mitochondrial cytochrome *b* gene sequences (13; Sakuma et al., unpublished data) (**b**). A median joining network is shown for haplotypes belonging to the mitochondrial subgroup CAS-1, which is thought to have experienced rapid expansion, perhaps associated with human activities related to agricultural development [5]. The scale bar shown below the tree represents genetic distance (**c**). The next range extension of the CAS-1 lineage is related to the CAS-1a group expansion, perhaps correlating with the spread of rice cultivation from South China to the Japanese Islands and the southern part of Sakhalin Island

The evolution of the genus *Mus* has involved phases of rapid speciation followed by allopatric divergence [1, 2]. Therefore, the area of origin for *M. musculus* must be a region that was not historically occupied by closely related species with similar ecological features, specifically other species of the Palearctic group (*M. musculus* species group: *M. spretus*, *M. macedonicus*, and *M. spicilegus*) and the Indian group (*M. booduga* species group: *M. booduga*, *M. terricolor*, etc.) [2]. These constraints restrict the candidate area to the region encompassing Iran, Afghanistan, Pakistan, and northern India. This is consistent with the homeland inferred from population genetic studies using mitochondrial DNA (mtDNA) (Fig. 1a). The earliest emerged lineages are the most restricted phylogroups, confined to the Arabian Peninsula [3] and the Himalayan region [4], providing robust evidence for the long-term residence of *M. musculus* in these geographic areas [5]. CAS consists of four mtDNA sublineages (CAS-1 to −4) that originated in the late Middle to Late Pleistocene (100,000–200,000 years ago), with a trend of confined distribution ranges around the eastern part of the Middle East. The exception is CAS-1, which appears to have spread rapidly during prehistoric times (e.g., 8,000 years ago), from a source region somewhere in India to the far eastern periphery of the CAS territory, including southern China [5]. Taking the early divergent sublineages of CAS mtDNA (CAS-2, −3, and −4) into account, the region of southwestern Asia encompassing modern day Iraq, Iran, Afghanistan, Pakistan, and northwestern India stands out as the most likely candidate area for the *M. musculus* homeland [3–10].

Great mtDNA diversity is seen in CAS populations inhabiting the mountainous region of northwestern India and Pakistan, while loss of haplotype diversity is evident from north to south on the Indian continent. This is indicative of a relatively recent range expansion of a unique lineage (CAS-1) to large geographic areas including the southern and eastern Indian subcontinent, Southeast Asia, Indonesia, southern China, northeastern China, and the Russian Far East (Fig. 1; [5, 7, 11, 12]). The geographical distribution of gene variation reflects the consequences of either random genetic drift or natural selection following genetic hybridization between geographic groups, resulting in gene-specific distribution patterns. Keeping this in mind, it is tempting to speculate that the southern and eastern parts of the Indian subcontinent are the sites of a secondary, but still somewhat ancient, distribution of *M. musculus*. Our recent studies on nuclear gene sequences revealed the presence of south India-specific phylogenetic elements, supporting the hypothesis that *M. musculus* settled long ago (e.g., 0.5 million years ago) in the eastern and southern parts of the Indian subcontinent [13], prior to the arrival of prehistoric humans. It is important to note that extensive genetic exchanges among the predicted geographic groups of CAS and among the three subspecies groups during the course of evolution are evident in haplotype analyses of linked nuclear genes [13].

Considering the current distribution of the mtDNA haplotypes and assuming that certain physical barriers have defined the borders of the three subspecies groups, one may tentatively define the range of *M. musculus* that existed before disturbances triggered by activities in the last 50,000 years by prehistoric humans (Fig. 1). The boundaries of the three major groups, CAS, DOM, and MUS, are demarcated by major geographic barriers [3, 5, 10]. The Zagros Mountains divide DOM in the west from CAS in the east, and the Elburz Mountains divide MUS in the north from CAS in the south. The mountain chains of the Hindu Kush separate populations of MUS and CAS in northern Afghanistan. Despite this information, the identity of the points of origin of the subspecies DOM and MUS remain unclear [12].

*M. musculus* has evolved to comprise three phylogroups, CAS, DOM, and MUS. The time of phylogroup divergence possibly dates back to the time of the divergence of *Mus musculus* from *M. spretus*, *M. spicilegus*, and *M. macedonicus* [2]. The CAS phylogroup is made up of several genetically distinct geographic groups in the predicted homeland area, including Pakistan and India. One of the prominent evolutionary features of this species is genetic exchange among the three subspecies and among the CAS geographic subgroups [10, 13].

### Two distinct radiation events are associated with the eastward movement of CAS

It was recently suggested that CAS mice experienced two rapid expansion and range extension events associated with the movements of prehistoric humans [5]. The date of the initial expansion event is calculated to be 7,600 years ago, using the tau (τ) value of 1.7 obtained from the *Cytb* sequence (1,140 bp) data for the mtDNA sub-lineage CAS-1 and an assumed value of 10%/site/million years for the evolutionary rate [5]. As has been postulated for the Middle East [14–16], it is possible to link this rapid expansion of the mouse population to certain historic human events. It is thought that trade networks dealing in domesticated cereal crops, including rice and millet, were established by about 9,000 years ago in several parts of South and East Asia [17–21]. Thus, it is plausible to link the rapid expansion of the CAS-1 mtDNA sequence data with agricultural development in the southeastern part of Asia, perhaps originating from somewhere in the Indian subcontinent, where CAS-1 mtDNA haplotypes now dominate (e.g., the northeastern area). It is possible to identify the extremeness of the rapid expansion, based on the presence of CAS-1 mtDNA in northeast China and the southern parts of the Russian Far East [5].

Notably, the CAS-1 group includes a subgroup (CAS-1a; see ref. [5]) that experienced rapid expansion as a separate, subsequent historical event. The presence of the locally restricted phyletic group CAS-1a is suggestive of stepwise historical range expansion for CAS-1 (Fig. 1). The descendant mtDNA haplotypes of the second expansion event are now found in parts of southern China (represented by Guilin and Kunming), northern Honshu, Hokkaido, and southern Sakhalin. It is thought that rice cultivation originated along the upper Yangtze River 4,500 years ago [22–24]. A recent extensive genome survey suggests that the Pearl River in southern China is the best candidate location for the first development of rice cultivation [24]. Thus, it is reasonable to speculate that the CAS-1a mtDNA subgroup expanded its range in association with the spread of rice culture from southern China to a wide area of East Asia, including the Japanese Islands [5, 25].

### Two distinct source areas for MUS suggest the existence of subspecies groups denoted “*musculus*” and “*wagneri*”

**Fig. 2:**
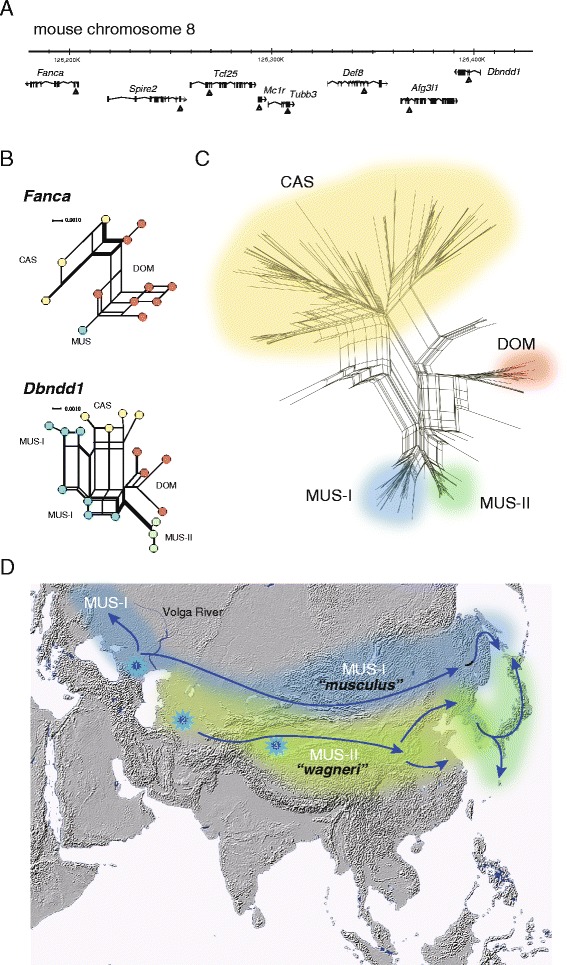
Nucleotide sequences of eight linked genes on mouse chromosome 8 were determined using wild mouse specimens representing the three major subspecies groups of *M. m. domesticus* (DOM), *M. m. castaneus* (CAS), and *M. m. musculus* (MUS) mice from Eurasia [13, 25] (**a**). Median joining networks of the *Fanca* and *Dbndd1* genes are shown as representative examples of low and highly divergent states, respectively, of the subspecies group (MUS) (**b**). A neighbor-net network with concatenated sequences (n = 196) exhibits clusters of the three major subspecies groups and a variety of recombinant haplotypes [13] (**c**). The two putative phylogroups of MUS are designated MUS-I and MUS-II [25]. A schematic view of the geographic subdivisions of the *Mus musculus musculus* house mouse subspecies groups (MUS) (**d**). The subdivision of the house mouse into the tentatively designated phylogroups *musculus* and *wagneri* has been inferred from morphological, ecological, cytogenetic, electrophoretic, and molecular studies (e.g. [25, 28–31]). Three possible source areas of the human-associated prehistoric dispersals of MUS are marked with star symbols: 1) Transcaucasia, 2) Turkmenistan/Kazakhstan, and 3) the Taklamakan Desert (see text)

There is a common perception that the MUS subspecies group has a unique genetic constitution, with a predicted evolutionary history that originated from a single source area and dispersed to the west and east in the northern Eurasian regions [7, 26, 27]. Our recent studies on tandemly linked nuclear gene sequences, however, provide robust evidence that the MUS subspecies group can be divided into two subgroups that localize to 1) northern (MUS-I) or 2) southern (MUS-II) parts of the MUS territory (Fig. 2) [25]. The chromosome region defined by the eight gene loci can be separated into two segments; one is a unique region, accounting for six loci, including *Fanca*, found in both MUS-I and MUS-II with low nucleotide diversity. In contrast, in the remaining two loci, *Afg3l1* and *Dbndd1*, the MUS-I related sequences exhibit a highly polymorphic state and apparent divergence from the MUS-II sequences, which are less polymorphic [25]. The low levels of genetic diversity of the upper chromosome segment can be explained by genetic introgression between the two geographically distinct MUS subgroups at a relatively recent time, but prior to their human-mediated long-distance dispersals. The contrasting patterns in the genetic diversity of the other two loci may reflect the ancestral state of genetic variation within each of the MUS subgroups. Although the evolutionary history of the *M. m. musculus* subspecies is complex and largely unknown, several pieces of evidence are available that enable us to reconstruct specific evolutionary episodes. The concept of a MUS homeland with two distinct lineages with an ancient onset of divergence is supported by both traditional and recent molecular studies, including those of a morphological, cytogenetic, and ecological nature (e.g., [28–32]).

The species and subspecies lineages related to *M. musculus* can be categorized into four distinct groups, based on morphological and ecological characteristics [28, 33], together with wild (outdoor) forms of 1) *M. m. wagneri*, 2) *M. m. manchu*, 3) *M. spicilegus*, and 4) *M. spretus*. The former three are associated with their representative commensal (indoor) forms of *M. m. castaneus/domesticus*, *M. m. molossinus* (the Japanese form), and *M. m. musculus*, respectively. Schwarz and Schwarz [28] reported that *wagneri* mice are distributed from the Volga River in the west to the Yellow Sea between 44°S and 36°S, and share a large territory in the northern part of Eurasia with its eastern neighbor, the *manchu* mice. The commensal “*musculus*” mice have a larger body and long tail, compared to the smaller body and short tail characteristic of “*wagner*” mice with “wild” behavior [28]. The average body weight of “*Mus wagneri*” is two-thirds that of “*Mus musculu*s” [34]. Mice occurring in the western (*musculus*) and eastern (*wagneri* and *manchu*) parts of the MUS territory can, therefore, be said to differ substantially in terms of both morphological and ecological characteristics. We believe that the molecularly based subgroups MUS-I and MUS-II localize to the western (or northern; *musculus*) and eastern (or southern; *manchu* and *wagneri*) parts of the MUS territory, respectively.

Studying cytogenetic variation in the pattern of C-banding provides insight into the spatial distribution of the MUS subspecies group. The CAS and DOM subspecies groups possess medium-sized heterochromatic blocks across the 20 pairs of chromosomes, including chromosome X. MUS mice, however, show a remarkable degree of polymorphism that correlates with geography. Mice from Europe exhibit a C-banding pattern similar to that seen in CAS and DOM, whereas mice from Asia exhibit a strikingly different genome constitution, wherein more than half of the autosomes and X-chromosomes are C-banding negative and some chromosomes possess large heterochromatic blocks and are designated as marker chromosomes [35–39]. This implies that the large or absent heterochromatic blocks are derived patterns, and therefore are likely to have emerged in an Asian portion of the MUS range. Taking into account the C-banding patterns, the simplest explanation is that the two divergent MUS cytogenetic groups originated from two different source areas located somewhere in central Asia or nearby. It is plausible that the karyotype groups with C-banding-positive and -negative patterns represent the aforementioned phylogroups of MUS-I (*musculus*) and MUS-II (*wagneri* and *manchu*), respectively.

Based on the karyological characteristics of the C-banding patterns, the eastern MUS group (*wagneri*) mice can be further divided into three distinct subgroups that appear to also differ in terms of geographic range. We tentatively denote the C-band-group subgroups as “*wagneri*” (no marker chromosomes), “*gansuensis*” (marker chromosomes 17^++^, 18^++^), and “*manchu*” (marker chromosome 18^++^). These subgroups can be distinguished by the presence or absence of the extra large sized C-banding (^++^) on the pericentromeric region of some chromosomes in the majority of individuals. Further studies are needed to elucidate the evolutionary episodes that resulted in the spatial variation with respect to the marker chromosomes.

Valuable information regarding MUS mice has been obtained from electrophoretic studies of β-globin (Hbb) and subsequent molecular studies on the corresponding gene (*Hbb*) (e.g., [6, 31, 40–44]). In *M. musculus* four major *Hbb* haplotypes, *d*, *p*, *s*, and *w1*, have been identified [31]. CAS is known to segregate into Hbb^*d*^ and Hbb^*p*^ haplotypes. Mice of the DOM subspecies group are associated with the Hbb^*d*^ and Hbb^*s*^ haplotypes. Both haplotypes are nearly always present at intermediate frequencies in populations of DOM from the Americas and Europe, and in some western populations of MUS located near the range boundary with DOM [45–47]. At the same time, Hbb^*s*^ is observed sporadically in MUS populations of northern Eurasia, from eastern Europe in the west to the Pacific Ocean in the east. Mice from the Asian territory of MUS possess the haplotypes Hbb^*d*^, Hbb^*p*^, and Hbb^*w1*^ [31, 42, 48, 49]. Hbb^*d*^ mainly occurs in populations of “north areas of MUS” from eastern Europe and Siberia. Hbb^*p*^ and Hbb^*w1*^ haplotypes prevail in populations of “south areas of MUS”, from central Asia (Turkmenistan, Uzbekistan, south east Kazakhstan) through to northern and eastern China. It is important to note that the central Chinese mice posses their own haplotype, Hbb^*w1*^. These observations support the concept of spatial subdivision of MUS mice into northern (MUS-I) and southern (MUS-II) phylogroups (Fig. 2).

In principle, the sharing of identical β-globin haplotypes among different taxa could be attributable to either introgressive hybridization or retention of ancestral polymorphism [50]. Introgressive hybridization is a plausible explanation for the sharing of identical Hbb alleles (or *Hbb* haplotypes) in natural populations between *M. m. castaneus* and *M. m. domesticus*, between *M. m. domesticus* and *M. m. musculus*, and between *M. m. domesticus* and *M. spretus* [26, 51–58]. Given the general nature of genetic exchanges among taxa before the historic dispersal events in this species, the shared haplotype of Hbb^*d*^ between the northern phylogroup of MUS (i.e., MUS-I) and CAS is likely attributable to historic genetic introgression, prior to the human-associated dispersal of MUS-I. It is noteworthy that Hbb^*p*^ and Hbb^*w1*^ tend to be confined to specific geographic areas of central Asia and northern and western China and the different genetic elements in these regions compared to the remaining territory of MUS, namely eastern Europe and Siberia, imply that the southern phylogroup MUS-II is more independent than the northern phylogroup MUS-I in terms of genetic distinctness from CAS and DOM. On the other hand, it is also necessary to consider the possibility of post-dispersal hybridization events between the northern and southern MUS lineages, since they share the Hbb haplotypes of their counterparts as minor elements. The geographical distribution of the *Hbb* haplotypes in MUS can be explained by either the invasion of *Hbb*^*p*^ and *Hbb*^*w1*^ mice into populations in the northern area or the converse invasion of *Hbb*^*d*^ mice into populations in the southern area associated with human settlement in central Asia [31].

Comparative studies of the gene and genome sequences of mice in the MUS territory provide evidence for the presence of at least two independent lineages, other than CAS and DOM [9, 30, 32, 50, 59, 60]. A recent study on mouse strains, including MSM/Ms, which originated from Japanese wild mice and therefore represents the MUS-II phylogroup (see ref. [25]), indicates that strains of MUS from Europe and East Asia possess substantially divergent genetic material [32].

Having established that MUS can be subdivided into MUS-I and MUS-II, we endeavored to predict the areas-of-origin of the two groups. These two MUS subgroups could represent the traditional taxa of *musculus* and *wagneri*. Schwarz and Schwarz [28] reported that the *wagneri* mice are distributed to the Volga River in the west. We can therefore assume that that the Caspian Sea and the Volga River demarcate the two groups. Transcaucasia is presumed to be the putative region where *M. m. musculus* diverged into subgroups [10, 12, 30, 61]. It follows from this that Transcaucasia can be considered the key source area of MUS-I (*musculus*) mice. The area-of-origin of MUS-II (*wagneri*) mice, however, is uncertain, although it is likely to be somewhere in central Asia, such as Turkmenistan, Kazakhstan or northwestern China (Fig. 2a). Eastern Kazakhstan and the regions south of the Taklamakan Desert are predicted to be the homeland of the *wagneri* mice, based on the predominant appearance of the *Hbb*^*w1*^ haplotype [52].

From analyses on mtDNA variation on wild mice from the large territory of MUS, including eastern Europe and East Asia achieved by the historic dispersal events [5], we can suggest that the initial expansion occurred 20,000–30,000 years ago. The initial expansion event can be explained by either in situ expansion at source areas of MUS prior to the historic long-range dispersal events or rapid acquisition of large territory along with the historic dispersal events. The secondary and more extensive expansion events were relatively recent, occurring 3,300–6,600 years ago in the northeasternmost part of the territory, including the Korean Peninsula (MUS-1c; see ref. [5]). It is likely that the evolutionary timeline of MUS expansion and divergence parallels certain anthropological movements, but the specific details are unknown. Careful attention to the archaeological record is needed to explore the history of the relationship between mice and humans [41].

The rapid expansion of the MUS-1 mtDNA haplotypes from Korea and Japan (Fig. 1b) is likely to be associated with the development of agricultural systems in the vicinity of the Korean Peninsula. The “MUS-1c” subgroup subsequently entered Japan from the Korean peninsula, perhaps a few thousand years ago [5], although we do not have any reliable genetic evidence to support this hypothesis [62]. Further research is required to elucidate the details of the predicted dispersals of wild mice that occurred in concert with historic events, such as the introduction of agriculture to the Japanese Islands and Korean Peninsula [5, 25].

## Conclusion

The recent extensive geographic sampling and mitochondrial and nuclear gene analyses allow us a finer view of the prehistoric dispersals to East Asia. We conclude that only one local lineage of CAS is involved in the long-range dispersal from the CAS homeland to wide areas of Southeast Asia, Indonesia, and continental East Asia, followed by a secondary expansion event that extended the CAS range from southern China to the insular part of East Asia, namely the Japanese Islands and southern Sakhalin. The subspecies group MUS is presumed to have generated two phylogroups, MUS-I and MUS-II, in its homeland somewhere near the Caspian Sea, fostering genetic exchanges between the phylogroups, prior to the long-range dispersals. The dispersals from the two different source areas have extended the territory of MUS to a large portion of northern Eurasia, from eastern Europe in the West to the Japanese Islands in the East. We tentatively assign the taxon names “*musculus*” and “*wagneri*” to the two phylogroups, which are associated with distinct genetic features characterized by morphological, chromosomal, biochemical, and nuclear genetic markers. Further studies are needed to confirm these hypotheses on the prehistoric eastward movements of CAS and MUS*.*
